# OpEx - a validated, automated pipeline optimised for clinical exome sequence analysis

**DOI:** 10.1038/srep31029

**Published:** 2016-08-03

**Authors:** Elise Ruark, Márton Münz, Matthew Clarke, Anthony Renwick, Emma Ramsay, Anna Elliott, Sheila Seal, Gerton Lunter, Nazneen Rahman

**Affiliations:** 1 The Institute of Cancer Research, London, Division of Genetics & Epidemiology, Sutton SM2 5NG, UK; 2 Wellcome Trust Centre for Human Genetics, Roosevelt Drive, Oxford OX3 7BN, UK; 3 Royal Marsden NHS Foundation Trust, Cancer Genetics Unit, Downs Road, Sutton, SM2 5PT, UK

## Abstract

We present an easy-to-use, open-source Optimised Exome analysis tool, OpEx (http://icr.ac.uk/opex) that accurately detects small-scale variation, including indels, to clinical standards. We evaluated OpEx performance with an experimentally validated dataset (the ICR142 NGS validation series), a large 1000 exome dataset (the ICR1000 UK exome series), and a clinical proband-parent trio dataset. The performance of OpEx for high-quality base substitutions and short indels in both small and large datasets is excellent, with overall sensitivity of 95%, specificity of 97% and low false detection rate (FDR) of 3%. Depending on the individual performance requirements the OpEx output allows one to optimise the inevitable trade-offs between sensitivity and specificity. For example, in the clinical setting one could permit a higher FDR and lower specificity to maximise sensitivity. In contexts where experimental validation is not possible, minimising the FDR and improving specificity may be a preferable trade-off for slightly lower sensitivity. OpEx is simple to install and use; the whole pipeline is run from a single command. OpEx is therefore well suited to the increasing research and clinical laboratories undertaking exome sequencing, particularly those without in-house dedicated bioinformatics expertise.

Exome sequencing is becoming a standard method used by increasingly diverse research and clinical laboratories. Unfortunately, easy-to-use, open-source exome analytical pipelines are not readily available, and most laboratories therefore implement bespoke pipelines^1^. There is wide variability in these underlying approaches and performance of analytical tools, and different versions of individual tools are often concurrently available, impeding standardisation and cross-validation. This is further exacerbated by the limited pipeline performance validation typically undertaken, particularly with respect to indel calling and specificity of pipelines, which are both vitally important for clinical exome sequencing. A further challenge for the clinical setting is the requirement to analyse large numbers of samples for optimal performance under the joint calling approach recommended by the most widely used software suite, GATK^2^. This is often not possible for clinical pipelines, where the need for fast test results necessitates analyses of smaller numbers of samples.

## OpEx Description

To address these issues we created the OpEx (Optimised Exome) analysis tool. OpEx includes a fixed implementation of tools for read alignment, variant calling and annotation optimised for individual or multiple exome sequencing analysis, outputting data to clinical standards. We specifically focused on making OpEx simple to install and to use; the whole pipeline is run with a single command. It is therefore well suited to the increasing number of research and clinical laboratories without in-house dedicated exome bioinformatic expertise. An overview of OpEx is given below. Full details of OpEx are given in the Supplementary Appendix and are available at www.icr.ac.uk/opex.

OpEx takes the raw FASTQ files generated by paired-end Illumina sequencing as its input, which are mapped to the human reference genome using Stampy with BWA for premapping^3^,^4^. A custom Python script then performs BAM sorting and indexing. Duplicate reads are marked with Picard (http://picard.sourceforge.net). We developed CoverView to provide the quality control information required in clinical laboratories. CoverView provides coverage, base quality and mapping quality metrics. CoverView utilises the user-supplied BED file and Ensembl transcripts to output the target regions which failed to achieve good quality coverage and annotates these regions according to their location in the gene transcript (e.g. c.1500–1550 of *BRCA1*). This seamless transcript-based annotation is particularly useful for the many laboratories that use orthogonal methods, such as Sanger sequencing, to ‘fill-in’ areas that fail to reach the required coverage threshold.

Within OpEx we provide a default transcript set which includes the Locus Reference Genomic (LRG) sequences to facilitate clinical utility^5^. However, users can specify any Ensembl transcripts, including the entire set of available transcripts, if preferred. For the default transcript we used BioMart to obtain a list of all known protein-coding transcripts. There were 83,331 different protein-coding transcripts corresponding to 20,228 genes. We selected the 19,922 genes present on chromosomes 1–22, X, or Y, that had both a start and stop codon, and were not known pseudogenes. 74% of these genes (n = 14,830) had more than one transcript. We selected a single transcript for these genes with preference for a CCDS transcript according to Supplementary Figure 1. The specific transcripts selected are given in Supplementary Table 1.

OpEx uses Platypus for variant calling^6^. Usefully, Platypus provides equivalent performance irrespective of the number of exomes being analysed. Analysis of many samples can thus be parallelised with no reduction in calling performance. Platypus also calls base substitutions and indels simultaneously. This rapid independent variant calling approach enables analysis to keep pace with the sequencing output, allowing early assessment of potential sample quality issues which are not detectable through the CoverView coverage metrics, e.g. a contaminated sample showing excess heterozygosity. It also provides early opportunities to assess and act on variant data, for example allowing early identification of a disease-causing variant in a plausible candidate gene. These give OpEx a useful advantage over callers that need to call across all samples together to provide optimal performance and thus cannot be run until all laboratory work for all exomes is complete. Of note, OpEx uses Platypus v0.1.5 for variant calling. We selected this version due to its mutually exclusive calling of a variant as either a base substitution or a simple insertion or deletion of sequence. Later versions of Platypus merge nearby base substitutions on the same allele into a single complex indel that does not change the length of the DNA sequence, requiring further bespoke code to recover the constituent variants for comparison with other data. Because the OpEx code is freely available, users have the flexibility to use different versions of any OpEx software component, including other versions of Platypus. However, it cannot be assumed that other versions will have exactly the same performance as reported here.

OpEx next uses CAVA to annotate variants^7^. Importantly, CAVA provides consistent annotation of indels, which is often suboptimal in annotation tools. Indel calling is challenging because there are often multiple equally correct ways of representing the indel in the standard VCF format exported from next generation sequencing (NGS) pipelines, many of which are inconsistent with clinical annotation conventions^7^. CAVA consistently provides the clinically appropriate indel call. Additionally, if an indel has an alternative representation, the most 3′ and the most 5′ annotations are outputted to facilitate appropriate investigation of the functional and/or clinical consequences.

Finally OpEx performs a parsing step using a custom Python script which assigns a high/low quality flag, removes intergenic calls, and returns an easily readable tab-separated output. Base substitutions are flagged as high quality if the Platypus QUAL score is at least 100. Indels are flagged as high quality if both the variant allele proportion (calculated from the TR/TC) is at least 0.2 and the Platypus FILTER value is PASS. OpEx then returns a simple tab-separated text file containing all variants occurring in the user-supplied transcripts.

OpEx provides standardised variant and coverage files but also allows flexibility for users to customise the default settings. OpEx also outputs useful interim files, such as the BAM file and the raw VCF file. Additionally, OpEx can output the variant file in VCF, allowing further downstream annotation, for example, with tools that predict variant functional/clinical impact^8^,^9^,^10^.

### OpEx evaluation

To evaluate OpEx performance we first analysed the ICR142 NGS validation series, which includes exome sequence FASTQ files for 142 samples together with Sanger sequencing data at 730 sites^11^. OpEx was run using one CPU per exome. Because samples are analysed independently, runtimes are limited only by the number of CPUs available and the size of the FASTQ file. The 142 samples had an average of 70 M reads in the input FASTQ files (range 30 M–159 M), resulting in an overall average runtime of 35 hours (range 17–101 hours). All 142 were run simultaneously, thus the actual runtime for the series was 101 hours. Faster runtimes would be possible with multithreading. Alignment constituted the majority of the runtime (69–88%), followed by the CoverView coverage evaluation (6–18%), though this could be reduced if evaluation is restricted to select genes, a common approach in clinical exome analysis. Variant calling and annotation comprised a very small proportion of runtime (0.4–1.6%) due to the rapid performance of Platypus and CAVA.

The ICR142 NGS validation series contains Sanger sequencing data for both positive and negative sites, with a particular focus on indels, allowing assessment of specificity as well as sensitivity for indels. Four of the 730 variant sites with Sanger data were in transcripts not included in the default OpEx database and were excluded. All of the remaining 726 sites had at least 15x coverage in the exome data, defined as at least 15 reads of good mapping quality (mapping score ≥ 20). We classified base substitutions as detected if the exact base change was called by OpEx. For indels, we also included the seven complex indels that were called by OpEx but annotated slightly differently from the Sanger call as having been detected. The evaluated sites are given in Supplementary Table 2. Length of indel impacted performance. For short indels, which we define here as being ≤10 bp, the sensitivity was 96% (258/269) and the false detection rate (FDR) was 6% (15/273). However, for longer indels, >10 bp, the FDR was much higher at 50% (16/32) though the sensitivity remained high 94% (16/17). As there were so few examples of this rare variant class with which to evaluate performance, we excluded indels >10 bp in our performance evaluation below, in which ‘all calls’ refers to all base substitutions and indels ≤10 bp.

The overall sensitivity of all calls was 97%, specificity was 90% and FDR was 8%. Using only the high-quality calls improved the specificity (97%) and FDR (3%) with a marginal reduction of sensitivity (95%). Sensitivity for base substitutions was excellent in both the all call set and the high-quality call set, but the specificity was markedly improved in the high-quality call set (from 60% to 96%). The indel calling performance was good, with overall sensitivity of 96%, specificity of 95% and FDR of 6% in the all call set. Using only high-quality calls, specificity (97%) and FDR (4%) of indel calls were improved, but there was some reduction in sensitivity (93%) (Table 1).

The eleven missed indels were explicable by the sequence context or quality: four were deletions in long homopolymer tracts, four were in regions with poor mapping quality, two had >25% of reads with poor base quality, and one was in a simple repeat (Supplementary Table 3). Thus it is possible to predict, and potentially flag, regions in which indel calling may be suboptimal, due to well-recognised limitations of short-read NGS data. Importantly, the nine false indel calls in the high-quality call set are all present in both the Exome Variant Server and ExAC databases, suggesting they may represent pipeline-independent artefacts of NGS analysis^12^,^13^.

Depending on the individual performance requirements the OpEx output allows one to optimise the inevitable trade-offs between sensitivity and specificity. For example, in the clinical setting one often tolerates a higher FDR and lower specificity to maximise sensitivity. In contexts where experimental validation is not possible, minimising the FDR and improving specificity is often an acceptable trade-off for slightly lower sensitivity.

We next applied OpEx to a large-scale dataset, the ICR1000 UK exome series, to better evaluate OpEx performance in detection of rare variation^14^. We selected 116 sites at which OpEx detected a variant in only one individual in the ICR1000 UK exome series, to evaluate performance at rare variation. Variant sites were validated by Sanger sequencing. The sites included 31 base substitutions, 36 deletions and 49 insertions. The 31 base substitutions, 13 of the deletions, and two of the insertions occurred in genes for which we already had Sanger sequencing primers in-house. The remaining 23 deletions and 47 insertions were selected randomly from amongst 96 individuals whose DNA was present on the same plate, to aid the laboratory work. OpEx performance was again excellent, with 97% sensitivity (31/31 base substitutions, 35/36 deletions, 47/49 insertions) and FDR of 3% (0/31 base substitutions, 1/36 deletions, 2/49 insertions). The three false positive variants were explicable by poor data quality: two were insertions of 6 bp and 8 bp present at the end of reads (a common site of false positive calls) and one was a 3 bp deletion in a region with poor mapping quality and poor coverage.

Platypus’s superior FDR in comparison with popular variant callers with similar sensitivities, such as GATK and samtools, has been previously reported^6^. The excellent sensitivities and FDRs we observed in both the ICR1000 and ICR142 series further support these data. Additionally, use of CAVA returned exactly matching annotations for indels when compared with the Sanger data, an advantage over other popular annotation tools, as previously described^7^.

Finally we tested OpEx performance in a proband-parent trio exome sequencing dataset to detect *de novo* variation, one of the commonest uses of clinical exome sequencing^15^. We used exome data from a trio in which the proband has an overgrowth syndrome due to a *de novo* indel mutation c.933_934insTCTT in *DNMT3A*^16^. We used a Python script to first identify all variant sites in the proband which were not present in either parent and then generated the coverage at these sites in both parents using CoverView. A variant was a candidate *de novo* variant if the quality flag was “high”, the TC value was at least 15x, and the coverage was at least 15x in both parents. Protein-altering variants were defined as those with CAVA class NSY, EE, IF, ESS, SG, or FS and those variants with CAVA class ESS, SG, or FS were further defined as protein-truncating^7^. Comparison of the proband and parental data highlighted 27 candidate *de novo* protein-altering variants, of which only two were protein-truncating variants, one of which was the *DNMT3A* mutation. The analysis was completed in 33 hours.

### Summary

OpEx is simple to install and can be run with a single command and input FASTQ files. The software is open source and freely available for download at www.icr.ac.uk/opex or at https://github.com/RahmanTeam/OpEx. The individual components of the pipeline are also freely available. Our detailed documentation, the availability of the OpEx source code, and the availability of the ICR142 and ICR1000 datasets allow users of other open-source frameworks to fully replicate and evaluate our pipeline in-house. However, we believe the primary advantage of OpEx is as a fully developed validated pipeline requiring minimal user input and no specialised informatic expertise. Due to its excellent performance and suitability for clinical and research settings, OpEx is now our standard pipeline for all exome analyses.

## Additional Information

**How to cite this article**: Ruark, E. *et al.* OpEx - a validated, automated pipeline optimised for clinical exome sequence analysis. *Sci. Rep.* **6**, 31029; doi: 10.1038/srep31029 (2016).

## Supplementary Material

Supplementary Information

Supplementary Dataset 1

## Figures and Tables

**Table 1:** OpEx variant calling performance in ICR142 NGS validation series.

	Variant type	All calls	High quality calls
Sensitivity	Overall*	378/389 (97%)	368/389 (95%)
	Base substitutions	120/120 (100%)	117/120 (98%)
	All indels	274/286 (96%)	266/286 (93%)
	Short indels	258/269 (96%)	251/269 (93%)
	Long indels	16/17 (94%)	15/17 (88%)
Specificity	Overall*	288/320 (90%)	309/320 (97%)
	No base substitution present	27/45 (61%)	43/45 (96%)
	No indel present (short only)	261/275 (95%)	266/275 (97%)
	No indel present (short and long)	245/275 (89%)	260/275 (95%)
False detection rate	Overall*	32/410 (8%)	11/379 (3%)
	Base substitutions	18/138 (13%)	2/119 (2%)
	All indels	32/307 (10%)	15/281 (5%)
	Short indels	15/273 (6%)	9/260 (4%)
	Long indels	16/32 (50%)	6/21 (29%)

^*^Overall numbers exclude long indels.
