# Supplementary material for: OpEx - a validated, automated pipeline optimised for clinical exome sequence analysis
Source: Sci Rep. 2016 Aug 3;6:31029. doi: 10.1038/srep31029 (PMC4971567; doi:10.1038/srep31029)
Supplement: Supplementary Information [file srep31029-s1.pdf]

OpEx - a validated, automated pipeline optimised for clinical exome sequence analysis

Elise Ruark[1], Márton Münz[2], Matthew Clarke[1], Anthony Renwick[1], Emma Ramsay[1], Anna Elliott[1], Sheila Seal[1], Gerton Lunter[2] and Nazneen Rahman[1,3*]

[1]The Institute of Cancer Research, London, Division of Genetics & Epidemiology, Sutton SM2 5NG, UK

[2]Wellcome Trust Centre for Human Genetics, Roosevelt Drive, Oxford OX3 7BN, UK

[3]Royal Marsden NHS Foundation Trust, Cancer Genetics Unit, Downs Road, Sutton, SM2 5PT, UK

***Corresponding Author:**

Professor Nazneen Rahman
The Institute of Cancer Research, London
15 Cotswold Road
Sutton SM2 5NG
United Kingdom
Email: rahmanlab@icr.ac.uk

Supplementary Figure 1: Flow diagram of default OpEx transcript selection per gene.

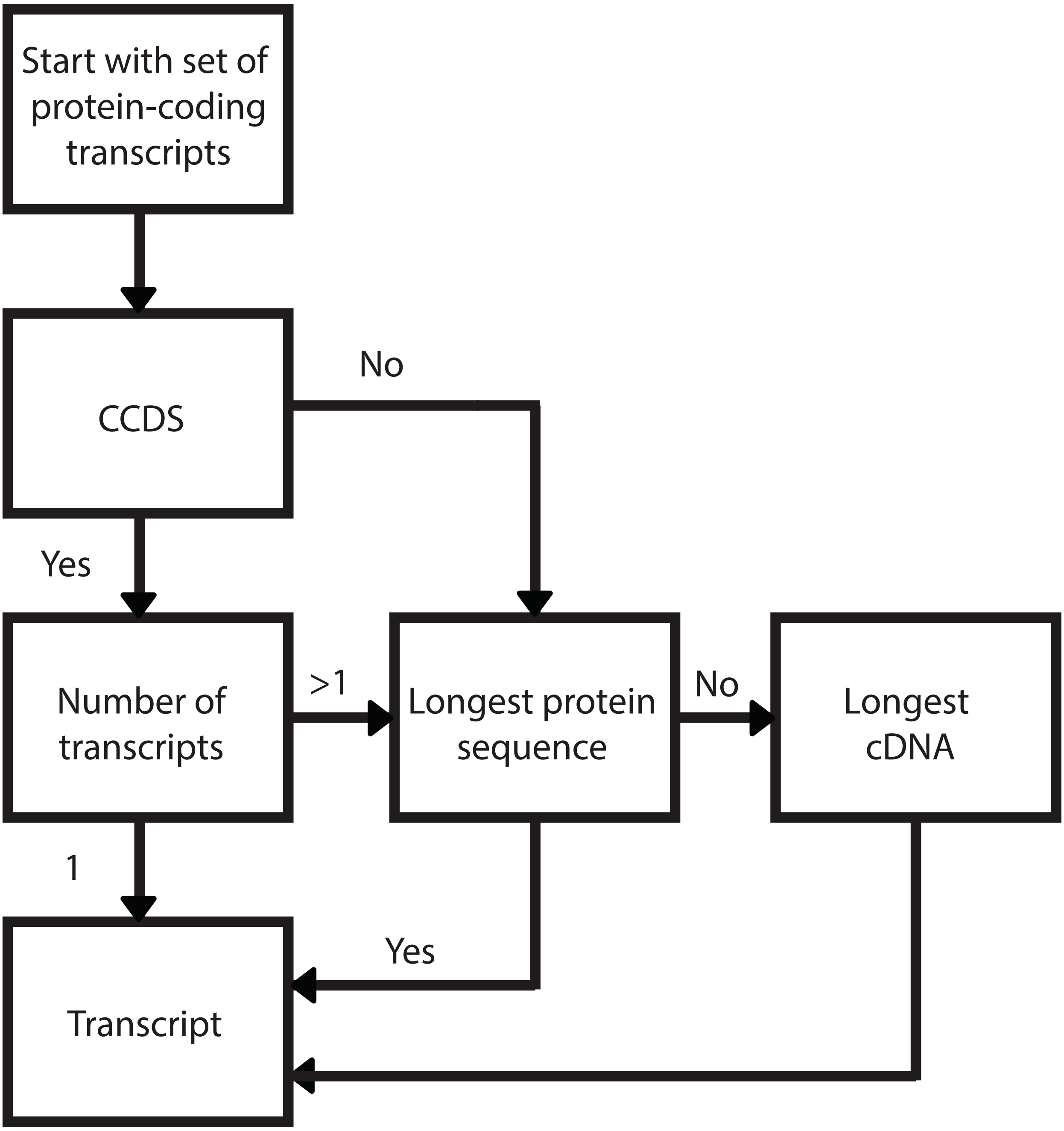

A single transcript was chosen manually for each gene according to the diagram above and used for all variants overlapping that gene. There were eight exceptions (*BRCA1*, *CDKN2A*, *CHEK2*, *FANCB*, *MEN1*, *MUTYH*, *TP53*, *TSHR*) where an alternative transcript was chosen on the basis of clinical relevance and literature review.

OpEx v1.0.0

Documentation

1 Introduction -------------------- Page 2
2 Minimum system requirements----- Page 3
3 Installation guide -------------- Page 4
4 Running OpEx -------------------- Page 7
5 Input files --------------------- Page 8
6 Output files -------------------- Page 9
7 Advanced settings --------------- Page 13
8 Contact ------------------------- Page 16

1 INTRODUCTION

The OpEx (Optimised Exome) pipeline has been developed through a collaboration between the Rahman group at the Institute of Cancer Research, London and the Lunter group at the Wellcome Trust Centre for Human Genetics, Oxford. The pipeline includes a fixed implementation of read alignment, variant calling and annotation tools optimized for individual or multiple exome sequencing analysis in the research or clinical setting.

OpEx can be installed by running a simple installation script with a single command, which automatically builds the entire pipeline (*see Section 3*).

OpEx can be run with a single command and is run independently at the sample level, providing equivalent performance for each sample, irrespective of the number of samples analyzed.

The pipeline takes the raw gzipped FASTQ files provided by paired-end Illumina sequencing as its input and generates a number of output files including read alignments, quality control metrics and annotated variant calls (see detailed description of input and output files in *Sections 5 and 6*). As its final output, OpEx provides a tab-delimited text file reporting all clinically annotated variant calls in a clear, simple-to-parse tabular format.

OpEx uses Stampy v1.0.14 for short read mapping, BWA v0.5.10 for premapping, Picard v1.48 for duplicate read marking, CoverView v1.1.0 for quality control analysis, Platypus v0.1.5 for variant calling and CAVA v1.1.1 for variant annotation (*see flowchart below*).

The default settings of OpEx are optimized for exome data analysis so the pipeline does not need to be configured; it is ready to run after installation. All its components (Stampy, Picard, CoverView, Platypus and CAVA) are run with their default settings. Furthermore, CAVA uses its default whole exome transcript database for variant annotation that is created based on Ensembl release version 65 and the human reference genome GRCh37.

However, the pipeline is customizable with a number of options available in its configuration files for advanced users (*see Section 7*) including setting the reference genome manually and changing the transcript database.

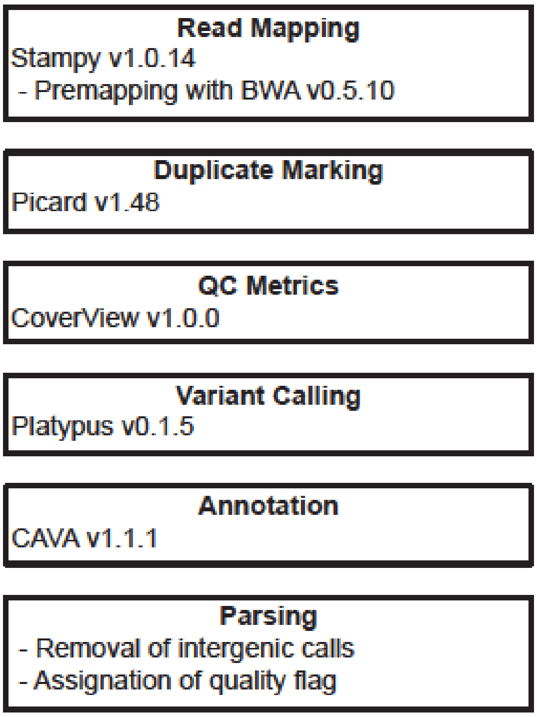

Flowchart summarizing the constituent steps of the OpEx pipeline

Recommended usage of OpEx:

- Run the pipeline for your paired-end Illumina data
- Monitor the run by looking at the log file. In the event of a hardware or software failure, error messages are printed to standard output
- Once OpEx has finished, check the reported quality control metrics to see if sequencing and read mapping were successful for each region of interest
- To double check any QC issues, you can go back to the BAM file
- Use the tab-delimited output file of annotated variant calls for downstream analysis
- To double check any variant call, you can go back to the VCF file

2 MINIMUM SYSTEM REQUIREMENTS

OpEx runs on Linux. It requires Python 2.7.3 or later (< Python 3) with Numpy version 1.11.0 installed and Java 1.6. At least 3 Gb of memory is required, and 8Gb is recommended. In order to make use of the optional multithreading feature, OpEx requires a multicore CPU environment.

3 INSTALLATION GUIDE

OpEx can be downloaded from http://www.icr.ac.uk/opex.

To install OpEx, unpack the tgz file and run the installation script (install.py) in the opex-v1.0.0 directory (see details below).

3.1 What will installation do?

The installation script will perform the following steps:

- Download all required components of the pipeline (i.e. BWA, Stampy, Picard, Platypus, and CAVA)
- Build all required components
- Index the reference genome (if given) by BWA and Stampy
- Generate the necessary default configuration files

3.2 Full Installation

In order to set up the pipeline correctly, we recommend running **Full Installation**. In **Full Installation**, the GRCh37 reference genome file has to be provided when running the installation script. The reference genome file will be automatically indexed by BWA and Stampy upon installation and therefore it can take a while (approx. 2-3 hours). There is also a **Quick Installation** option (*Section 3.3)*.

Go into the opex-v1.0.0 folder and run:

./install.py -r /path/to/reference/human_g1k_v37.fasta

where human_g1k_v37.fasta is the file of the GRCh37 reference genome sequence which (together with the corresponding .fai file) can be downloaded from: ftp://ftp.1000genomes.ebi.ac.uk/vol1/ftp/technical/reference/

Note that the .fai file will also need to be in the same folder as the .fasta file.

Once the installation script has finished, OpEx is ready for use.

If issues arise during automatic installation, please see the **Manual Installation** option in *Section 3.4*.

3.3 Quick Installation

In **Quick Installation** one is not required to provide the reference genome, instead a path pointing to an existing genome installation can be set manually (see *Section 7.4*) or the reference can be supplied upon the first run.

Go into the opex-v1.0.0 folder and run:

./install.py

Once the installation script has finished, OpEx is ready for use. However, the GRCh37 reference genome must be set manually or supplied upon first run.

3.4 Manual installation

The OpEx pipeline can be installed in a fully automatic way by running the install.py script (see above). However, if issues arise during automatic installation, the pipeline can also be installed manually:

1. First, execute all commands in the **build_opex.sh** bash script. *This script downloads and builds all required components of the pipeline.*

2. Create a **config.txt** file in the opex-v1.0.0 folder and add the following lines with the appropriate paths:

   ENSTDB = /path/to/opex-v1.0.0/exome_65_GRCh37.gz
   CAVA_CONFIG = /path/to/opex-v1.0.0/cava_config.txt
   GENOME_INDEX = /path/to/opex-v1.0.0/index/ref
   HASH = /path/to/opex-v1.0.0/index/ref
   REFERENCE = /path/to/reference/human_g1k_v37.fasta

   This step is required to create the main configuration file of OpEx.

3. Copy the **templates/coverview_config_template** file as **CoverView_default.json** into the opex-v1.0.0/ folder and add the following lines to the end of the file:

   "only_fail_profiles": true,
   “transcript”: {“regions”: false, “profiles”: false, “poor”: true},
   "transcript_db": "/path/to/opex-v1.0.0/exome_65_GRCh37.gz" }

4. Copy the **templates/coverview_config_template** file as **CoverView_full.json** into the opex-v1.0.0/ folder and add the following line to the end of the file:

   “transcript”: {“regions”: true, “profiles”: true, “poor”: true},
   "transcript_db": "/path/to/opex-v1.0.0/exome_65_GRCh37.gz" }

Step 3 and 4 are required to create the CoverView configuration files.

5. Copy the **templates/cava_config_template** file as **cava_config.txt** into the opex-v1.0.0/ folder and add the following lines to the end of the file:

   @ensembl = /path/to/opex-v1.0.0/exome_65_GRCh37.gz
   @reference = /path/to/reference/human_g1k_v37.fasta

   This step will create the CAVA configuration file.

6. Finally, execute all commands in the **index_genome.sh** bash script. *This script will run BWA and Stampy to index the reference genome.*

3.5 Testing installation

A test dataset is included with the package to confirm OpEx is installed correctly. The test dataset consists of:

- **Input test files**:
  Two gzipped FASTQ files (test_R1.fastq.gz, test_R2.fastq.gz) containing 372 read pairs mapping to three exons of *BRCA2* and a BED file (test.bed) containing the coding exons of *BRCA2* in hg19 genomic coordinates.

- **Expected test output files**:
  Eleven output files (as described in Section 6) generated by a correct installation of OpEx. Four files (the bash script file, the log file, the Picard metrics file, and the Platypus log file) are not included as these are dependent on the date, time, and system and are thus not informative as a test of successful installation.

The test dataset and the expected outputs are found in the test/ and test/output/ directories, respectively.

To test the installation, go into the opex-v1.0.0 folder and run:

./test_installation.py

Due to the small size of the test dataset, the test script typically finishes in less than a minute. If the test script reports "*OpEx is correctly installed*", the pipeline was successfully run from beginning to end on the test dataset, the outputs agree with the expected outputs, and resulting outputs are removed. If the test of the installation is not successful, the script reports "*OpEx is not installed correctly*" and the resulting outputs are placed in a folder called _testinstall for manual inspection.

4 RUNNING OPEX

Run OpEx from any directory with the following command:

/path/to/opex-v1.0.0/opex.py -i R1.fastq.gz,R2.fastq.gz -b panel.bed -o sample1

The -i (or --input) option specifies the comma-separated paired-end gzipped FASTQ files (R1/R2) used as the input of the pipeline.

The -b (or --bed) option specifies a BED file defining regions of interest. Note that the -b flag is optional. If it is not given, only a chromosome level coverage summary will be outputted. If a BED file is provided, coverage metrics will be evaluated for the regions specified in the BED file (see *Section 6.4*).

The -o (--output) option specifies an arbitrary sample identifier which is used as prefix for the output file names.

Optionally, the -k (--keep) command line flag allows the user to retain all temporary files created by the pipeline, which are automatically removed by default.

4.1 Specifying reference genome at first run

If the reference genome was not provided upon installation or set manually, it has to be specified using the -r (or --reference) option when first running OpEx (but is not required at later runs):

opex.py -i R1.fastq.gz,R2.fastq.gz -o sample1 –r /path/to/reference/human_g1k_v37.fasta

4.2 Multithreading

Two components of the OpEx pipeline (CoverView and CAVA) have inbuilt multithreading features that allow running of both the quality control analysis and variant annotation as multiple parallel processes, when multiple CPU cores are available. This can reduce the total runtime of OpEx.

Multithreading is optional and can be switched on with the -t (or --threads) command line flag, specifying the number of processes to be used. For example, in order to use 4 parallel processes:

opex.py -i R1.fastq.gz,R2.fastq.gz -o sample1 -t 4

4.3 Extended output information

Using the OpEx command line option -f (or --full), CoverView will provide additional output information (see *Section 6.4* for more details):

opex.py -i R1.fastq.gz,R2.fastq.gz -o sample1 -b exome.bed -f

Note that using this option will result in increased runtime.

In order to achieve the fastest running speed for large BED files such as the whole exome, it is recommended to use both the default setting (i.e. -f flag not switched on) and option -t (multithreading). For example:

opex.py -i R1.fastq.gz,R2.fastq.gz -o sample1 -b exome.bed -t 4

4.4 Custom configuration file

By default, OpEx uses the configuration file created automatically by the installation process and located in the /path/to/opex-v1.0.0/ folder (config.txt). Alternatively, a custom configuration file can be supplied by using the command line option -c. For example:

opex.py -i R1.fastq.gz,R2.fastq.gz -o sample1 -b exome.bed -c myconfig.txt

See Section 7 for the list of advanced settings that are customizable in the configuration files.

5 INPUT FILES

As discussed above, the input files for OpEx are as follows:

- Two gzipped FASTQ files (R1 and R2) with reads from paired-end Illumina sequencing of a single sample.
- A .BED file following the format described on the UCSC Genome Bioinformatics web site: http://genome.ucsc.edu/FAQ/FAQformat with each record corresponding to a region of interest (e.g. exon). The BED file is required to be sorted by the chromosome field and is assumed 0-based.

6 OUTPUT FILES

OpEx generates 15 output files for each sample:

6.1 General output files

- **Bash script file (<name>_opex_pipeline.sh)**:
  This automatically generated bash script file is the main script executed in the OpEx pipeline. It also serves as a documentation of the exact steps performed to analyze the sample and is kept for future reference.

- **OpEx log file (<name>_opex_log.txt)**:
  Status information on the current OpEx run. If a software or hardware failure is encountered, the "Pipeline finished" message is not present at the end of this file.

6.2 Stampy output files

- **a BAM file and BAM index (.bai) file (<name>.bam)**:
  Mapped short reads outputted by Stampy

6.3 Picard output files

- **a BAM file and BAM index (bai) file (<name>_picard.bam)**:
  Mapped short reads after duplicate marking by Picard

- **Picard metrics file (<name>_picard_metrics.txt):**
  Text file given by Picard reporting duplication metrics

6.4 CoverView output files

CoverView v1.1.0 reports QC results in multiple files with increasing levels of detail from a chromosome level summary to per-base profiles. It also flags regions that fail pre-defined quality requirements.

The four CoverView output files are as follows:

- **Chromosome level summary file (<name>_coverview_summary.txt):**
This output file gives a chromosome level summary of coverage. Each chromosome is described in a separate line with the following four columns:

1. **Chromosome name**
2. **Total read count (RC)**: total number of reads mapped to the chromosome

3. **Read count in regions (RCIN)**: total number of reads mapping to the chromosome that overlap regions from the BED file
4. **Read count outside of regions (RCOUT)**: total number of reads mapping to the chromosome that do not overlap regions from the BED file

In addition to the list of chromosomes, the outputted table also reports the mapped, unmapped and total read counts for the whole dataset.

- **Per-base profiles for failed regions (<name>_coverview_profiles.txt):**

By default, per-base profiles for each failed region are reported in this output file (see the definition of a 'failed region' below). Each position is described in a separate line with the following 8 columns:

1. **Chromosome**
2. **Position**
3. **Coverage (COV)**: number of reads covering the position
4. **Quality coverage (QCOV)**: number of reads covering the position with a read mapping quality >20 and a mapping base with base quality >10
5. **Median base quality (MEDBQ)**: median base quality of all read bases mapping to the position
6. **Fraction of low base quality (FLBQ)**: fraction of read bases mapping to the position with a base quality <=10
7. **Median mapping quality (MEDMQ)**: median mapping quality of all reads covering the position
8. **Fraction of low mapping quality (FLMQ)**: fraction of reads covering the position with a mapping quality <=20

- **Summary metrics for all regions (<name>_coverview_regions.txt):**

This output file provides a number of different metrics summarizing the per-base profiles of each region. These summary metrics give information on the overall quality of each region. In addition, regions are marked as 'PASS' or 'FAIL'. Each line in the file corresponds to a region described by the following 12 columns:

1. **Region name**
2. **Chromosome**
3. **Start position of region**
4. **End position of region**
5. **'PASS' or 'FAIL'**: The region is flagged 'FAIL' if MINQCOV<15 and 'PASS' otherwise
6. **Read count (RC)**: Total number of reads overlapping with the region
7. **Median coverage (MEDCOV)**: Median of coverage (COV) values across all positions in the region
8. **Minimum coverage (MINCOV)**: Minimum of coverage (COV) values across all positions in the region
9. **Median quality coverage (MEDQCOV)**: Median of quality coverage (QCOV) values across all positions in the region
10. **Minimum quality coverage (MINQCOV)**: Minimum of quality coverage (QCOV) values across all positions in the region

11. **Maximum fraction of low mapping quality (MAXFLMQ)**: Maximum of FLMQ values across all positions in the region
12. **Maximum fraction of low base quality (MAXFLBQ)**: Maximum of FLBQ values across all positions in the region

Note that the MEDCOV, MINCOV, MEDQCOV, MINQCOV, MAXFLMQ and MAXFLBQ values are derived from the per-base COV, QCOV, FLMQ and FLBQ profiles defined above.

The region name in the first column is taken from the 4th column of the BED file. If there are multiple regions in the BED file with the same name in their 4th column (e.g. the regions correspond to different exons of the same gene), CoverView adds an index to the region names joined by an underscore. For example, multiple regions of the *BRCA2* gene would be referred to as BRCA2_1, BRCA2_2, BRCA2_3, etc.

The above cutoff values for mapping and base quality and the requirement for a region to be flagged as 'FAIL' are set in the OpEx default settings, but can be customized; see *Section 7.1*.

- **Poor quality ranges within regions (<name>_coverview_poor.txt):**

This output file provides a comprehensive list of all continuous ranges within the regions of interest where QCOV<15 for all bases (referred to as 'poor quality' ranges). Note that multiple such ranges may exist in a single region. Each line in the file corresponds to a 'poor quality' range with the following 6 columns:

1. **Region name**: name of region incorporating the range
2. **Chromosome**
3. **Start position of range**
4. **End position of range**
5. **Transcript start position**: start position mapped to c. transcript coordinate(s) in the corresponding Ensembl transcript(s), if any
6. **Transcript end position**: end position mapped to c. transcript coordinate(s) in the corresponding Ensembl transcript(s), if any

Note that the Ensembl transcripts overlapping with the region are obtained from the default whole exome transcript database also used by CAVA.

The user can also choose not to supply a BED file, in which case only the _coverview_summary.txt file will be outputted and the last two columns (RCIN and RCOUT) will not be included in the file.

The user can also choose to generate full output information by specifying the –f command line option flag, returning the following additional information:

- The transcript coordinates are also given in the _coverview_regions.txt and _coverview_profiles.txt files:

    - in the _coverview_regions.txt file: start and end positions of each region mapped to c. transcript coordinate(s) in the corresponding Ensembl transcript(s), if any
    - in the _coverview_profiles.txt file: every position mapped to c. transcript coordinate(s) in the corresponding Ensembl transcript(s), if any

- Per-base profiles are outputted for all regions and not only for failed regions in the _coverview_profiles.txt file

6.5 Platypus output files

- **VCF file (<name>_calls.vcf):**
  Variant calls reported by Platypus

- **log file (<name>_platypus_log.txt):**
  Original Platypus log file providing status information on variant calling

For more details on outputs, please refer to the Platypus documentation:
http://www.well.ox.ac.uk/platypus-doc

6.6 CAVA output file

- **annotated VCF file (<name>_annotated_calls.vcf):**
  Variant calls annotated by CAVA, added to the INFO field of the VCF file

For the variant annotations reported by CAVA and output syntax, please refer to the CAVA v1.1.1 documentation:
http://www.icr.ac.uk/cava

6.7 Final OpEx output file

- **a tab-delimited .txt file (<name>_annotated_calls.txt):**
  annotated variant calls reported in a tabular format; each line corresponds to a single variant with the following 23 columns:

1. Chromosome (CHROM)
2. Position (POS)
3. Reference allele (REF)
4. Alternative allele (ALT)
5. Quality score (QUAL) - see Platypus documentation (linked above)
6. Quality flag (QUALFLAG): Value of "high" if the variant is a base substitution with QUAL score of 100 or higher, or the variant is an indel with a variant allele proportion (as defined by the TR value divided by the

TC value) greater than 0.2 and the variant has a FILTER value of PASS. Value of "low" otherwise.
7. Variant calling filter (FILTER) – see Platypus documentation
8. Total number of reads containing the variant (TR) – see Platypus documentation
9. Total coverage at this locus (TC) – see Platypus documentation
10. Sample name (SAMPLE)
11. Genotype called in the sample (GT) – see Platypus documentation
12. Variant type (TYPE) – see CAVA documentation (linked above)
13. Ensembl transcript ID (ENST) – see CAVA documentation
14. Gene symbol (GENE) – see CAVA documentation
15. Transcript information (TRINFO) – see CAVA documentation
16. Within-transcript location of variant (LOC) – see CAVA documentation
17. Clinical Sequencing Nomenclature (CSN) annotation – see CAVA documentation
18. Variant class annotation (CLASS) – see CAVA documentation
19. Sequence ontology annotation (SO) – see CAVA documentation
20. Variant impact (IMPACT) – see CAVA documentation
21. Alternative annotation (ALTANN) – see CAVA documentation
22. Alternative CLASS annotation (ALTCLASS) – see CAVA documentation
23. Alternative SO annotation (ALTSO) – see CAVA documentation

Only variant calls that overlap a gene transcript are reported in this file. Platypus v0.1.5 reports complex indel variants (those involving more than a simple insertion or deletion of sequence) as a simple indel and a nearby base substitution on the same allele, denoted with the same genotype.

Note that unlike in the VCF format, annotation information for multiallelic variant calls are split to multiple lines. If a variant call overlaps multiple transcripts, the information is also split into multiple records: each line represents annotations with regard to a different transcript (see examples in the CAVA documentation.)

7 ADVANCED SETTINGS

The OpEx pipeline is configured with the default settings of its components. One can, however, easily customize some components such as CoverView and CAVA by changing the configuration files.

7.1 CoverView settings

CoverView v1.1.0 uses a configuration file of JSON format. The OpEx installation creates two CoverView configuration files in the opex-v1.0.0/ folder;

CoverView_default.json and **CoverView_full.json**, that are used to generate the default and extended output, respectively.

Users can create their own configuration file (e.g. **custom.json**) and supply it to OpEx in the following way:

1. Start running OpEx in the usual way, then terminate it
2. In the generated _opex_pipeline.sh Bash script, change **CoverView_default.json** to **custom.json**
3. Re-run the _opex_pipeline.sh script

For an example of the JSON structure of the configuration file, see **CoverView_default**. Possible options are the following:

- **"duplicates"** (Boolean): if true, duplicate reads are included in the analysis

- **"outputs"** (JSON object): can contain the following two fields:
  - "regions" (Boolean): if true, the _regions.txt output file is written
  - "profiles" (Boolean): if true, the _profiles.txt output file is written

- **"low_bq"** (integer): base quality cut-off used in the FLBQ metrics

- **"low_mq"** (integer): mapping quality cut-off used in the FLMQ metrics

- **"transcript_db"** (String): path to transcript database file (if not given, transcript coordinates are not reported in the output files)

- **"transcript"** (JSON object): can contain the following three fields:
  - "regions" (Boolean): if true, transcript coordinates are reported in the _regions.txt output file
  - "profiles" (Boolean): if true, transcript coordinates are reported in the _profiles.txt output file
  - "poor" (Boolean): if true, transcript coordinates are reported in the _poor.txt output file

- **"only_fail_profiles"** (Boolean): if true, only failed regions are outputted in the _profiles.txt file

- **"fail"** (JSON object): requirements for a region to pass (fail)
  Can contain the following number fields:
  - "MIN_MINCOV": minimum value allowed for the MINCOV metrics
  - "MIN_MEDCOV": minimum value allowed for the MEDCOV metrics
  - "MIN_MINQCOV": minimum value allowed for the MINQCOV metrics
  - "MIN_MEDQCOV": minimum value allowed for the MEDQCOV metrics
  - "MAX_MAXFLMQ": maximum value allowed for the MAXFLMQ metrics
  - "MAX_MAXFLBQ": maximum value allowed for the MAXFLBQ metrics

For instance, if **“fail”: {“MINQCOV”: 15, “MAXFLMQ”: 0.2}** is used, regions that have MINQCOV<15 or MAXFLMQ>0.2 are defined as failed regions.

7.2 CAVA settings

The OpEx installation creates a CAVA configuration file in the opex-v1.0.0/ folder, **cava_config.txt**. Users can create their own configuration file (e,g. **cava_config_custom.txt**) and supply it to OpEx the following way:

1. Change the CAVA_CONFIG setting in the main OpEx configuration file (**config.txt** in the opex-v1.0.0 folder or custom config file used by the -c command line flag) from **cava_config.txt** to **cava_config_custom.txt**

For the complete list of settings available in the CAVA configuration file, please refer to the CAVA v1.1.1 documentation:
http://www.icr.ac.uk/cava

7.3 Using a different transcript database

By default, CoverView and CAVA use the same transcript database (**exome_65_GRCh37.gz**). Users can supply a custom transcript database (e.g. **custom_transcripts.gz**) generated by the CAVA dbprep tool (see CAVA documentation). Changing the transcript database for OpEx involves the following steps:

1. Copy the **custom_transcripts.gz** and **custom_transcripts.gz.tbi** files into the opex-v1.0.0/ folder
2. Change the ENSTDB setting in the main OpEx configuration file (**config.txt** in the opex-v1.0.0 /folder or custom config file used by the -c command line flag) from **exome_65_GRCh37.gz** to **custom_transcripts.gz**
3. Change the @ensembl setting in the CAVA configuration file (**cava_config.txt**) from **exome_65_GRCh37.gz** to **custom_transcripts.gz**.

7.4 Setting the reference genome files manually

Either upon installation or at the first run, OpEx requires the user to index the reference genome file with BWA and Stampy. In case the user already has the GRCh37 reference genome indexed by the correct version of Stampy and/or BWA, the corresponding paths to the index/hash files can be manually added to the OpEx configuration file by the following steps:

1. Install OpEx with the Quick installation mode (see *Section 3.2*)

2. Add the following lines (with the appropriate paths) to the **config.txt** file in the opex-v1.0.0/ folder (or a custom config file used by the -c command line flag):

REFERENCE = /path/to/reference/human_g1k_v37.fasta
GENOME_INDEX = /path/to/Stampy/index/filename
HASH = /path/to/Stampy/hash/filename

(OpEx assumes that the BWA index files are in the same folder as the .fasta file.)

8 CONTACT

Please submit all bug reports, comments, questions and feature requests in the OpEx User Group on Google Groups:
https://groups.google.com/forum/#!forum/opex-user-group
Feedback can also be sent via email to opex-user-group@googlegroups.com.
